# Low-Voltage Area at the Anterior Wall of the Left Atrium Is Associated With Thromboembolism in Atrial Fibrillation Patients With a Low CHA_2_DS_2_-VA Score

**DOI:** 10.3389/fcvm.2022.869862

**Published:** 2022-06-13

**Authors:** Xiangwei Ding, Mingfang Li, Hongwu Chen, Gang Yang, Fengxiang Zhang, Weizhu Ju, Kai Gu, Jianqing Li, Minglong Chen

**Affiliations:** ^1^School of Biomedical Engineering and Informatics, Nanjing Medical University, Nanjing, China; ^2^Department of Cardiology, The First Affiliated Hospital of Nanjing Medical University, Nanjing, China; ^3^Department of Cardiology, Taizhou People's Hospital, Taizhou, China

**Keywords:** atrial fibrillation, left atrial low-voltage area, thromboembolism, risk factors, low-risk

## Abstract

**Background:**

Non-valvular atrial fibrillation (NVAF) in patients at low risk of thromboembolism (TE) does not mean “no risk.” We sought to assess the risk factors associated with TE in clinically low-risk AF patients with a non-gender CHA_2_DS_2_**-**VASc score (CHA_2_DS_2_-VA score) of 0 or 1.

**Methods:**

In this single-center cross-sectional study, NVAF patients with a CHA_2_D**-**VA score of 0 or 1 who underwent index high-density bipolar voltage mapping of the left atrium (LA) and AF ablation were consecutively enrolled from 2017 to 2020. The population was divided into patients with and without TE history before voltage mapping. AF patients with CHA_2_DS_2_-VA score of 0 to 1 before TE (TE group) were analyzed and compared with clinically low-risk AF patients without TE history (non-TE group). The association among LA low voltage area (LVA), other clinical factors and TE history was analyzed with logistic regression.

**Results:**

In the TE group, LVA was more prevalent [15/25 (60%) vs. 105/359 (29.2%), *p* = 0.003] and more preferentially located at the anterior wall [8/15 (53%) vs. 24/105 (23%), *p* = 0.025]. Among patients with LVA, the activation time from the sinus node to the left atrial appendage was significantly longer in the TE group (77.09 ± 21.09 vs. 57.59 ± 15.19 ms, *p* < 0.001). Multivariate analysis demonstrated that LVA at the anterior wall of the LA [OR: 4.17 (95% CI: 1.51 to 11.51); *p* = 0.006] and being female [OR: 3.40 (95% CI: 1.36 to 8.51); *p* = 0.009] were associated with TE history.

**Conclusions:**

LVA at the anterior wall of the LA is associated with TE history in NVAF patients with a low CHA_2_DS_2_-VA score.

## Introduction

Non-valvular atrial fibrillation (NVAF) increases the risk of stroke by 5 times (1). In addition, strokes associated with AF are usually more severe and more frequently lead to disability and death (2, 3). The CHA_2_DS_2_-VASc score is widely used for stroke risk stratification in patients with NVAF. However, a small proportion of AF patients with a low CHA_2_DS_2_-VASc score still develop thromboembolism (TE). They are often young with valuable social roles. Therefore, screening for other risk factors predictive of TE in AF patients with low CHA_2_DS_2_-VASc scores is essential.

Every comprehensive scoring system might not be perfect enough. Recently, some studies have demonstrated that the discrimination power of the CHA_2_DS_2_-VASc score in predicting TE in AF patients was only modest (4, 5). The most likely primary reason is that the CHA_2_DS_2_-VASc score is a vascular scoring system that does not incorporate AF-related parameters. It was reported that approximately 0.2% of low-risk AF patients classified by the CHA_2_DS_2_-VASc score still had an unexpected stroke (6). Since these patients have a low risk of stroke and are relatively young, AF-related strokes in this cohort have a significant social impact. However, very few studies have identified additional risk factors for TE in low-risk AF patients (7, 8). Therefore, the predictors of TE in this cohort remain uncertain. Left atrial fibrosis detected using cardiac magnetic resonance imaging (MRI) is thought to be associated with TE in the general NVAF population (9, 10). Whether left atrial fibrosis could predict TE in low-risk AF patients has not yet been reported. The left atrial bipolar low-voltage area (LVA) identified using three-dimensional (3D) electroanatomic mapping could be used as a surrogate marker of left atrial fibrosis (11–13). The aim of this study was to assess the association of left atrial LVA with a thromboembolic history in relatively low-risk AF patients.

## Methods

This was a single-center, cross-sectional observational study. The study protocol followed the principles of the Helsinki Declaration and was approved by the Human Research Ethics Committee of the First Affiliated Hospital of Nanjing Medical University. Written informed consent was signed by all patients. The data of this article will be shared on reasonable request to the corresponding author.

### Study Population

The presence of AF was identified with an electrocardiogram (ECG). The inclusion criteria were as follows: (1) patients aged 18 to 74 years; (2) non-valvular AF; (3) CHA_2_D**-**VA score of 0 or 1; and 4) patients undergoing 3D electroanatomic high-density bipolar voltage mapping of the left atrium (LA). The exclusion criteria were as follows: (1) previous AF ablation history; (2) patients with thrombi in the LA; (3) left atrial size ≥55 mm (2-dimensional transthoracic echocardiography); (4) patients with severe structural cardiac disease (severe heart valve regurgitation, hypertrophic cardiomyopathy, or other severe valvular disease); (5) congenital heart disease; or (6) patients with carotid or cerebral artery stenosis.

### Definition of the Explanatory Variables

The CHA_2_DS_2_-VASc score incorporates the patient's age, sex, and history of ischemic stroke/transient ischemic attack/systemic embolism, and the presence of congestive heart failure, hypertension, diabetes mellitus, and vascular disease. Since female sex is now considered a risk modifier instead of a risk factor for stroke in NVAF patients (7, 14), a non-gender CHA_2_DS_2_-VASc score (CHA_2_DS_2_-VA score) of 0 or 1 was defined as a clinically low risk of TE in our study. AF patients with CHA_2_DS_2_-VA scores of 0 to 1 before the thromboembolic event (TE group) were analyzed and compared with low-risk AF patients without TE history (the non-TE group). Paroxysmal AF (PAF) was defined as continuous AF for <7 days. Non-PAF was defined as continuous AF for >7 days, which included long-standing persistent AF for >1 year.

### Catheter Ablation Procedure and Mapping

Antiarrhythmic medications were withheld for 5 half-lives before the procedure. Transesophageal echocardiography or computed tomography scanning was performed to rule out left atrial thrombi for all patients preceding the procedure. After observing the left atrial geometry, pulmonary vein isolation (PVI) was performed under conscious sedation using intravenous fentanyl and via transseptal access to the left atrium. Assessment of LA surface area and LVA followed a clinical routine and were performed as described previously (15, 16). A sequential high-density 3D bipolar voltage map (at least 300 points per patient) was constructed in all patients during sinus rhythm, guided by a 3D electroanatomic mapping system (CARTO3, Biosense Webster, or Ensite velocity, Abbott) using a circular mapping catheter (Lasso, 4 mm interelectrode spacing, Biosense Webster or Advisor FL, 3 mm, interelectrode spacing, Abbott). Electrical cardioversion was performed when the patients were in AF. Stable contact between the local atrial tissue and the electrodes of the mapping catheter was required. Internal point filter software was used to limit data acquisition. Only mapping sites that were within a distance of 5 mm from the acquired LA shell contributed to the voltage map. LVA was defined as contiguous areas (at least 3 adjacent points) of bipolar voltage < 0.5 mV. Extra care was taken at the sites where LVA was recorded to exactly define the extent of LVA.

### Evaluation of LVA and Activation Time From the Sinoatrial Node (SN) to the Left Atrial Appendage (LAA)

**Figure 1:**
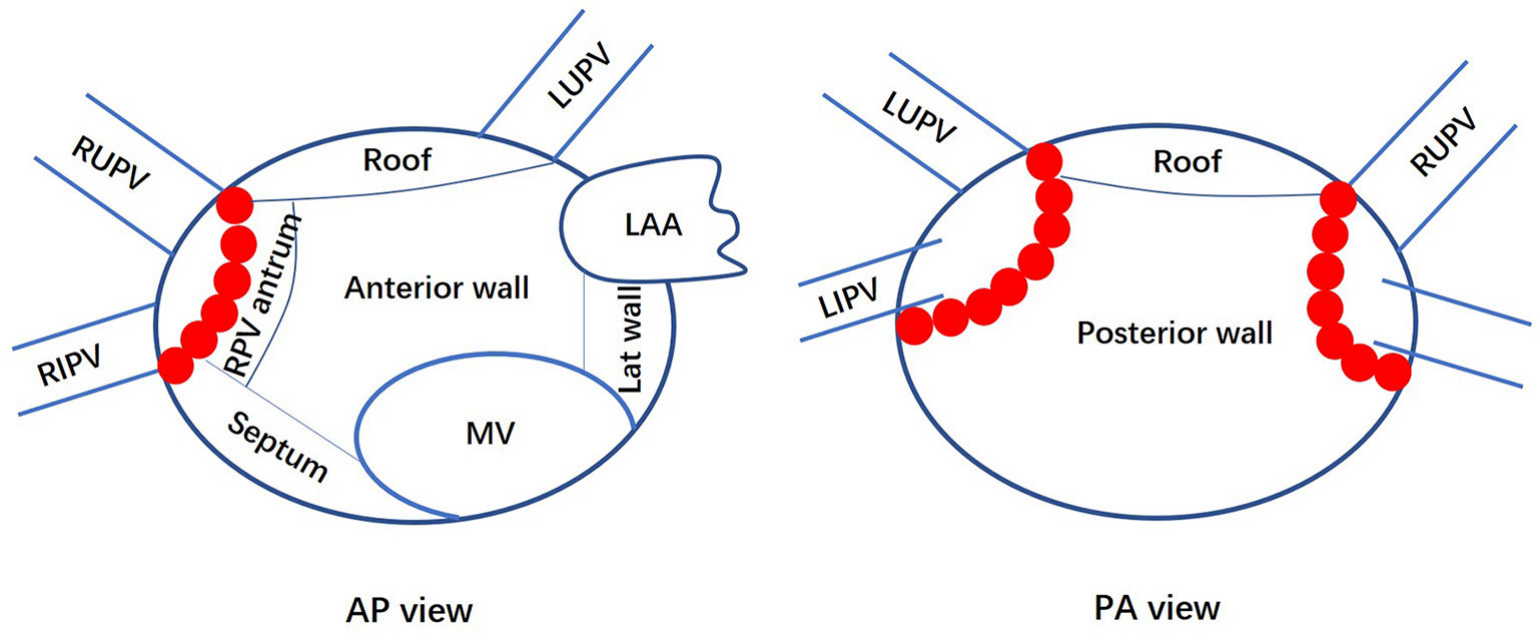
Anatomic divisions of the left atrium. Anteroposterior (AP) and posterior-anterior (PA) views of the left atrium show how it was divided for the analysis of the low voltage area distribution. LA, left atrium; MV, mitral valve; LAA, left atrial appendage; Lat, lateral; PV, pulmonary vein; RS, right superior; RI, right inferior; LS, left superior; LI, left inferior; RPV, right pulmonary vein. Red dots represent ablation points on the pulmonary vein isolation line.

The LA was segmented into six areas: anterior wall, posterior wall, right pulmonary vein antrum, roof, septum, and lateral wall (Figure 1). The distribution and surface area of the LVA were calculated. The ratio of the LVA to the LA area was defined as the percentage of the LA area within the isolation line, not including the ablation lesions in the surface area of the LA and anatomical structures such as the PV, LAA and mitral annulus. Among patients with left atrial LVA, the activation time from the SN to the LAA was also recorded by measuring the time from the onset of the P wave in Lead II of the surface ECG to the earliest activation of the LAA during sinus rhythm.

### Statistical Analysis

All analyses were performed using SPSS 19.0 statistical software. Normally distributed continuous variables are presented as the mean ± standard deviation (SD) and were compared between groups using Student's *t*-test. Categorical variables are presented as counts with percentages. The difference between groups was tested using the chi-square test. Fisher's exact test was performed for low expected counts (*n* <5). All parameters with a significance of *p* < 0.05 in the univariate analysis were entered into the multiple logistic regression analysis. The multivariate logistic regression method was used to determine the association of the clinical variables with the thromboembolic history. A *p*-value < 0.05 was considered statistically significant.

## Results

**Figure 2:**
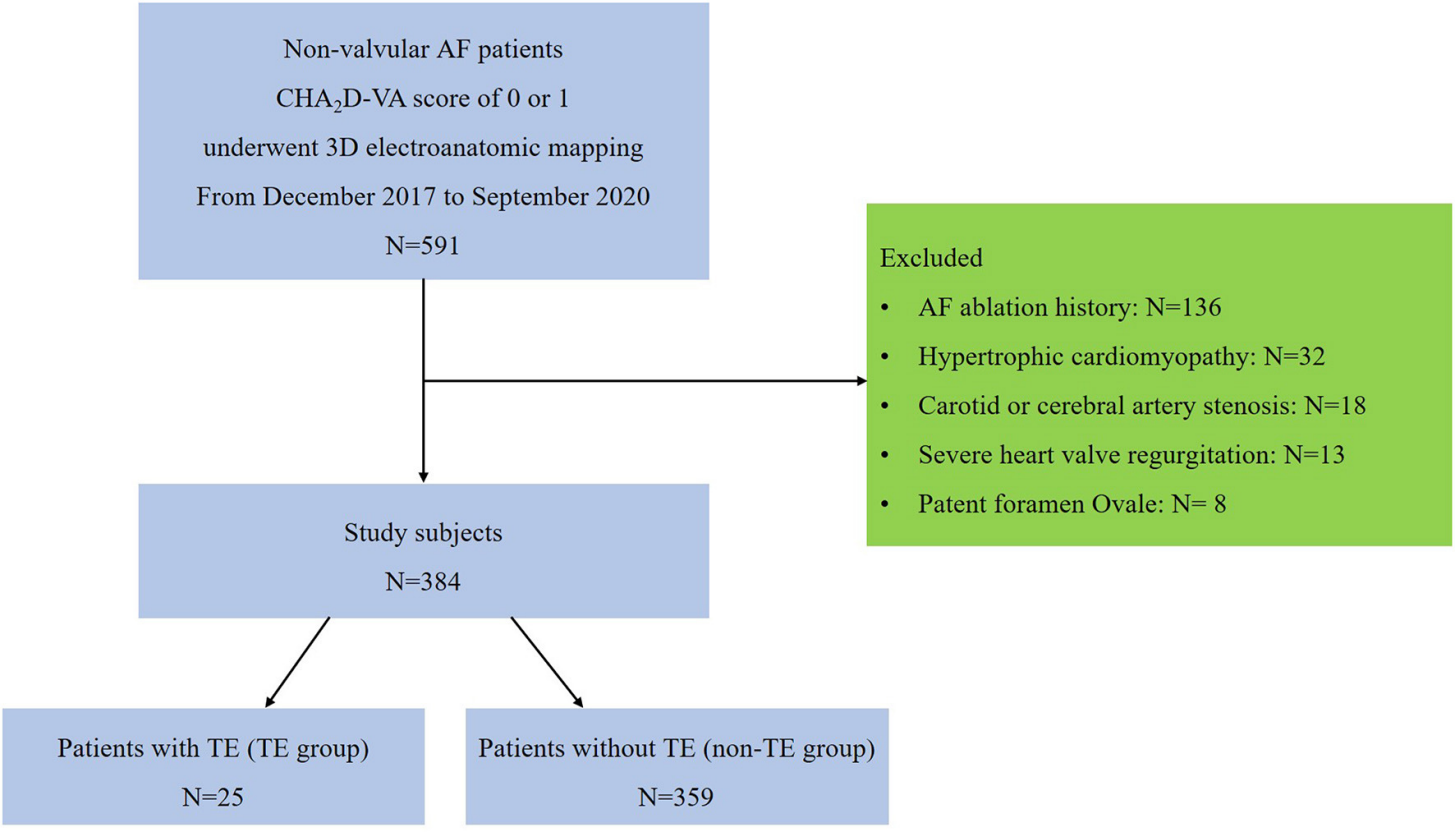
Flow diagram showing the process for selecting our study patients. AF, atrial fibrillation; TE, thromboembolism.

From December 2017 to September 2020, 591 consecutive NVAF patients with a CHA_2_D-VA score of 0 or 1 underwent 3D electroanatomic high-density bipolar voltage mapping of the LA in our center. After eliminating individuals who met the exclusion criteria, we finally enrolled 384 patients. Figure 2 shows the patient flow of this study.

According to whether there was a history of TE before the voltage mapping of the LA, the patients were divided into 2 groups: the TE group (*n* = 25) and the non-TE group (*n* = 359). The median time interval between the TE events and the voltage mapping of the LA was 10 months (IQR: 3–36 months). All patients in the TE group had CHA_2_DS_2_-VA scores of 0 to 1 and none was on anticoagulation before the first thromboembolic event occurred (23 patients with ischemic stroke, 1 patient with a left renal arterial embolism, and 1 patient with an arterial embolism in the lower right limb). Brain imaging studies were performed for all patients with ischemic stroke. The interpretation of the study results was validated by a neurologist. The etiology of the thromboembolic events in all 25 patients was considered to be cardioembolic and related to the AF.

Table 1 summarizes the baseline characteristics of 384 patients. In the entire cohort, the mean age was 56.4 ± 8.5 years, and 73.2% of the patients were men. Compared with patients in the non-TE group, those in the TE group were more likely to be women (60.0 vs. 24.6%, *p* < 0.001) and had higher levels of N-terminal pro brain natriuretic peptide (NT-pro BNP) (1200.04 ± 1049.00 vs. 840.91 ± 811.54 ng/L, *p* = 0.042). The CHA_2_D-VA score was similar between these two groups (0.60 ± 0.50 vs. 0.54 ± 0.50, *p* = 0.546). There was no significant difference regarding age, smoking status, type of AF, hypertension, hyperlipidemia, diabetes, heart failure, LA size (represented as LA anterior-posterior diameter) or left ventricular ejection fraction (LVEF) between these two groups.

**Table 1:** Baseline characteristics of the study population.

	**TE group**	**Non-TE group**	***P*** **-value**
**Number**	25	359	
**Demographics**			
Age (y)	59.0 ± 9.7	56.2 ± 8.4	0.116
Female sex	15 (60.0%)	88 (24.5%)	**<0.001**
Body mass index	26.08 ± 2.98	25.29 ± 2.68	0.183
Smoking	4 (16.0%)	101 (28.1%)	0.248
**History variables**			
Paroxysmal AF	9 (36.0%)	75 (20.9%)	0.084
Heart failure	0 (0%)	1 (0.3%)	1.000
Hypertension	11 (44.0%)	137 (38.2%)	0.672
Diabetes	0 (0.0%)	7 (1.9%)	1.000
Hyperlipidemia	4 (16.0%)	58 (16.2%)	1.000
CHADS_2_ score	0.44 ± 0.51*	0.40 ± 0.49	0.702
CHA_2_DS_2_-VA score	0.60 ± 0.50*	0.54 ± 0.50	0.546
**Echocardiography**			
LAD (mm)	41.52 ± 4.24	40.91 ± 4.12	0.480
LVDD (mm)	47.24 ± 4.19	48.59 ± 4.05	0.107
LVEF (%)	62.38 ± 3.96	61.83 ± 5.13	0.604
**NT-pro BNP (ng/L)**	1200.04 ± 1049.00	840.91 ± 811.54	**0.042**

* *, CHADS_2_ score and CHA_2_DS_2_-VA score before thromboembolic event. TE, thromboembolism; AF, atrial fibrillation; LAD, left atrial diameter; LVDD, left ventricular end diastolic dimension; LVEF, left ventricular ejection fractions; NT-pro BNP, N-terminal pro brain natriuretic peptide. Bold values meant statistically significant*.

### Left Atrial LVA and the Thromboembolic Events

Table 2 shows that left atrial LVA was more prevalent in the TE group than in the non-TE group (60.0 vs. 29.2%, *p* = 0.003). Among patients with left atrial LVA, the ratio of the LVA to LA area was similar between these two groups, but the activation time from SN to LAA was significantly longer in the TE group (77.09 ± 21.09 vs. 57.59 ± 15.19 ms, *p* < 0.001) (Table 2).

**Table 2:** The prevalence of left atrial LVA, ratio of left atrial LVA to LA area, and the activation time from SN to LAA.

	**TE group**	**Non-TE group**	***P*** **-value**
Number	25	359	
Left atrial LVA	15 (60.0%)	105 (29.2%)	**0.003**
Ratio of LVA to LA area (%)	12.40 ± 6.33	9.31 ± 6.31	0.118
The activation time from SN to LAA (ms)	77.09 ± 21.09	57.59 ± 15.19	**<0.001**

*LVA, low voltage area; LA, left atrium; SN, sinoatrial node; LAA, left atrial appendage. Bold values meant statistically significant*.

**Figure 3:**
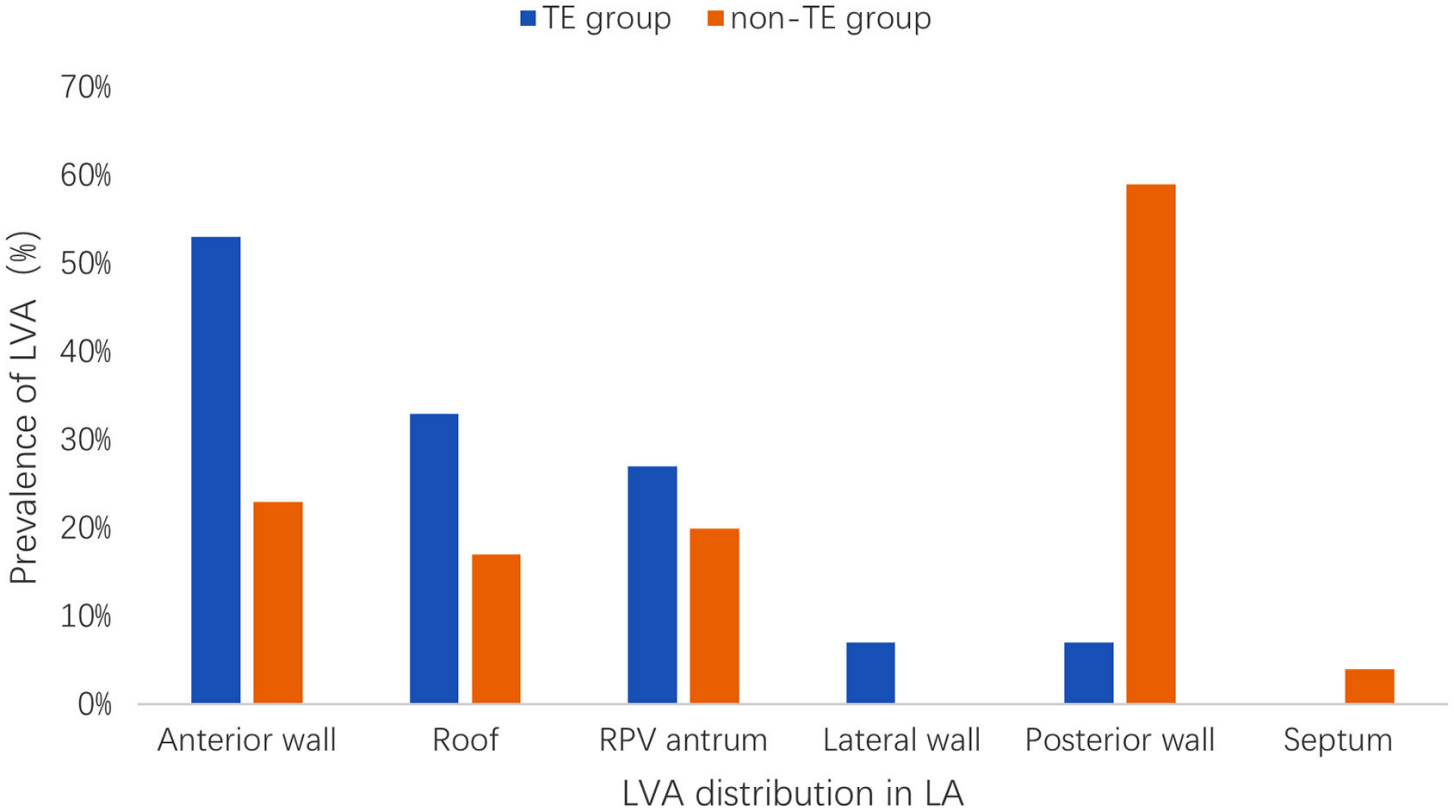
Distribution and prevalence of LVA in LA. LVA, low voltage area; LA, left atrium; TE, thromboembolism; RPV, right pulmonary vein.

Figure 3 depicts the distribution of LVA in the LA among all of the patients with left atrial LVA. In the TE group, 8 patients (53%) had a documented LVA on the left atrial anterior wall, 5 (33%) on the roof region, 4 (27%) on the right pulmonary vein antrum, 1 (7%) on the lateral wall, 1 (7%) on the posterior wall, and none on the septum. In the non-TE group, 24 patients (23%) had a documented LVA on the left atrial anterior wall, 18 (17%) on the roof region, 21 (20%) on the right pulmonary vein antrum, none on the lateral wall, 62 (59%) on the posterior wall, and 4 (4%) on the septum. The left atrial LVA was more preferentially located at the anterior wall in the TE group than in the non-TE group (53 vs. 23%, *p* = 0.025). The posterior wall was more frequently affected in the non-TE group than in the TE group (62 vs. 7%, *p* < 0.001).

In the univariate analysis, the existence of anterior LVA in the LA, female sex, and elevated levels of NT-pro BNP were significantly associated with a TE history. Multivariate analysis demonstrated that LVA at the anterior wall of the LA [OR: 4.17 (95% CI: 1.51 to 11.51); *p* = 0.006] and being female [OR: 3.40 (95% CI: 1.36 to 8.51); *p* = 0.009] were associated with a history of TE (Table 3).

**Table 3:** The logistic regression analysis to identify risk factors associated with TE.

	**Univariate analysis**		**Multivariate analysis**	
	**OR (95% CI)**	***P*** **-value**	**OR (95% CI)**	***P*** **-value**
Female sex	4.62 (2.00–10.65)	**<0.001**	3.40 (1.36–8.51)	**0.009**
NT-pro BNP	1.00 (1.00–1.01)	**0.049**	1.00 (1.00–1.01)	0.088
LVA at the anterior wall of LA	6.57 (2.57–16.76)	**<0.001**	4.17 (1.51–11.51)	**0.006**

*TE, thromboembolism; AF, atrial fibrillation; NT-pro BNP, N-terminal pro brain natriuretic peptide; LVA, low voltage area; LA, left atrium. Only parameters with a significance of p < 0.05 in the univariate analysis were listed in the table. Bold values meant statistically significant*.

## Discussion

In this cross-sectional observational study, we found that left atrial LVA was more prevalent in clinically low-risk AF patients with TE than in those without TE. In low-risk AF patients with TE, the left atrial LVA was preferentially located at the anterior wall. Conduction from the SN to the LAA is usually delayed compared with patients without TE. Moreover, LVA at the anterior wall of the LA and female sex were significantly associated with a thromboembolic history in clinically low-risk AF patients.

### Other Risk Factors in Addition to Those Included in the CHA_2_DS_2_-VASc Score

It has been speculated that some additional independent risk factors for AF-related TE may not be included in the CHA_2_DS_2_-VASc score (17). In the general NVAF population, several studies have identified additional risk factors (18–20), such as NT-pro BNP, left ventricular diastolic dysfunction, and impaired kidney function. However, in the low-risk AF population, very few studies have evaluated potential predictors of AF-related TE (7, 8). A retrospective, cross-sectional study showed that old age (≥60 years), high NT-pro BNP (≥300 pg/mL), low creatinine clearance (<50 mL/min), and an enlarged LAD (≥45 mm) were independently associated with stroke in clinically low-risk AF patients (7). Another matched case-control study revealed that smoking was a predictor of ischemic stroke in low-risk AF patients (8). In that study, some smokers in the non-stroke group might have been undetected because the patients without records on smoking were all considered non-smokers. This makes the conclusion of the study less compelling. Considering the significant social impact of stroke in these low-risk patients, it is essential to identify novel risk factors that can predict TE in this cohort.

### Left Atrial LVA, Female Sex, LA Fibrosis and Thromboembolic Events

In animal models, an increased LA size was associated with a higher degree of left atrial interstitial fibrosis (21, 22). A clinical study by Fatema et al. (23) reported that LA volume enlargement was a biomarker for ischemic stroke. However, since left atrial fibrosis may occur before apparent enlargement of the LA, echocardiographic measurement of the LA size may result in an underestimation of the association of LA fibrosis and thromboembolic events.

Daccarett et al. and Akoum et al. (9, 10) elegantly showed that a high degree of left atrial fibrosis, identified by DE-MRI, is independently associated with the left atrial thromboembolic milieu and a prior history of stroke in patients with AF. In addition, recent studies revealed that left atrial LVA (a bipolar voltage range of 0.1–0.5 mV), detected using 3D electroanatomic mapping during sinus rhythm and used as a surrogate marker of left atrial fibrosis, was associated with postprocedural silent cerebral events and a history of stroke (12, 13). These results are consistent with our findings of the association between left atrial LVA and TE. In our study, left atrial LVA was more prevalent in the TE group than in the non-TE group. However, unlike previous studies, the total prevalence of LVA was relatively low in our study. A major reason is that all patients enrolled in our study had few comorbidities. In addition, the catheters used in our study had a large inter-electrode gap, which reduced the accuracy of the maps and may partly explain the different results.

In our study, patients in the TE group were more likely to be women. Pfannmüller et al. (24) reported that fibrosis-related genes were upregulated in postmenopausal women with AF. A histological study analyzing atrial tissue obtained during cardiac surgery illustrated that women had higher expression of CX 40 than men, which indicated remodeling-induced changes in connexins (25). In our study, multivariate analysis demonstrated that being female was associated with LVA at the anterior wall of the LA (Table 4), and LVA at the anterior wall of LA was more prevalent in patients with a prior history of TE. However, based on our analysis, the prevalence of LVA at the posterior wall of the LA did not differ between male and female (15.3 vs. 19.4%, *p* = 0.352). Meanwhile, the increased LVA at the posterior wall in the non-TE group did not reach the significant difference (*p* = 0.097). Thus, being prone to fibrosis at the anterior wall of the LA may be an important reason to explain why female sex was a risk factor for TE in clinically low-risk AF patients in our study. However, considering that the association of female sex with higher risk of TE was independent of LVA at the anterior wall of the LA, the specific mechanism of the association between female sex and TE needs further investigation.

**Table 4:** The logistic regression analysis to identify risk factors associated with LVA at the anterior wall of LA.

	**Univariate**		**Multivariate model**	
	**OR (95% CI)**	***P*** **-value**	**OR (95% CI)**	***P*** **-value**
Age (y)	1.18 (1.11–1.26)	**<0.001**	1.17 (1.09–1.25)	**<0.001**
Female sex	5.40 (2.53–11.50)	**<0.001**	3.76 (1.70–8.33)	**0.001**

*LVA, low voltage area; LA, left atrium. Only parameters with a significance of p < 0.05 in the univariate analysis were listed in the table. Bold values meant statistically significant*.

### LVA at the Anterior Wall, Activation Time From SN to LAA, and Thromboembolic Events

It has been recognized that most emboli arise from the LAA in patients with AF (26). In our study, the left atrial LVA was more preferentially located at the anterior wall in the TE group than in the non-TE group. LA fibrosis existing in the anterior wall region has been shown to be correlated with a reduction in LAA flow velocity (27). While this reduction is strongly related to the presence of emboli in patients with AF. In our study, we additionally found that the activation time from the SN to the LAA was significantly longer in the TE group than in the non-TE group. This delayed conduction from the SN to the LAA, which was dominantly determined by the anterior wall fibrosis, could indirectly reflect a reduction in LAA flow velocity. Therefore, our findings partially provide additional plausible evidence for an association among anterior LA fibrosis, LAA function and thromboembolic events.

### LA Fibrosis and Thromboembolic Events

There is increasing evidence showing that AF may not be a prerequisite for embolic stroke (28, 29). Instead, AF could be considered as a manifestation of fibrotic atrial cardiomyopathy that has progressed to a certain stage (30, 31). The underlying mechanism of embolic stroke might be related to atrial fibrosis and atrial cardiomyopathy. Under such conditions, LA endothelial functional impairment might exist, with a probably abnormal coagulation state and delayed LAA emptying function. All of these factors could work together, and LAA clot formation might be the final consequence.

### Clinical Implications

Based on the results of this study, we propose that for clinically low-risk AF patients, especially for women, LVA at the anterior wall of the LA could be used to refine the risk stratification for the risk of TE and guide the anticoagulation strategy following PVI.

### Limitations

The inherent limitation of this study is its cross-sectional nature. Although we illustrated that LVA in the anterior wall of the LA and female sex were independently associated with a history of TE in clinically low-risk AF patients, further prospective studies are still needed. Second, it should be noted that LA voltage mapping was performed after the TE occurred. The delay between the TE events and the mapping varied. Furthermore, LA remodeling is a dynamic process, which may have an unknown effect on our observed finding. However, we reported here findings of a hypothesis-generating study that illustrated the association between LVA at the anterior wall of the LA and TE. Third, whether LVA detected by 3D electroanatomic voltage mapping could accurately reflect LA fibrosis is still debatable. Fourth, since the number of patients was relatively small, it was difficult to analyze the correlation among LAA 3D geometry, LAA Doppler flow velocity and TE, but this will be performed in our future study. Fifth, it seems to be an overstatement to conclude that the presence of low-voltage areas alone is associated with thrombotic risk. Other data, specifically serological data, information an any abnormal coagulation status and assessment of left atrial and left ventricular function by ultrasound, would also be required. Sixth, both recorded low voltage area and the intra-atrial conduction time were measured in sinus rhythm. Since it is in AF rhythm that thrombosis is actually more likely to occur, more functional mapping of the left atrium would be the preferred method of assessment. The novelty of this paper would be enhanced by the addition of different pacing sites and the evaluation of low potential areas and conduction times during rapid atrial pacing. Finally, this study was a single-center study. The study results might be hard to extrapolate to general NVAF patients with low CHA_2_DS_2_-VA scores.

## Conclusion

LVA at the anterior wall of the LA is associated with TE in clinically low-risk AF patients. This finding might be helpful for refining the risk stratification for the risk of TE in AF patients with low CHA_2_DS_2_-VA scores and for guiding the anticoagulation strategy after AF ablation.

## Author's Note

The corresponding author MC takes responsibility for all aspects of the reliability and freedom from bias of the data presented and their discussed interpretation.

## Data Availability Statement

The data of this article will be shared on reasonable request to the corresponding authors.

## Ethics Statement

The studies involving human participants were reviewed and approved by the Human Research Ethics Committee of the First Affiliated Hospital of Nanjing Medical University. The patients/participants provided their written informed consent to participate in this study. Written informed consent was obtained from the individual(s) for the publication of any potentially identifiable images or data included in this article.

## Author Contributions

MC had full access to all of the data in the study and takes responsibility for the integrity of the data and the accuracy of the data analysis. JL and MC contributed to the concept and design. XD, ML, HC, GY, FZ, WJ, KG, JL, and MC contributed to acquisition and analysis or interpretation of the data. XD and ML contributed to drafting of the manuscript and contributed to statistical analysis. XD, ML, JL, and MC contributed to critical revision of the manuscript for important intellectual content. All authors contributed to the article and approved the submitted version.

## Funding

This study was funded by the Hospital program for Innovative Research Teams (ID: IRT-004) and the Key Clinical Study Project of Jiangsu Province, China (ID: BE2017750). The funders are non-industrial organizations and had no role in the study design, study conduct, data analysis or interpretation, authorship decisions or paper publication. The corresponding author JL or MC had full access to all of the data and the final responsibility for the decision to submit for publication.

## Conflict of Interest

The authors declare that the research was conducted in the absence of any commercial or financial relationships that could be construed as a potential conflict of interest.

## Publisher's Note

All claims expressed in this article are solely those of the authors and do not necessarily represent those of their affiliated organizations, or those of the publisher, the editors and the reviewers. Any product that may be evaluated in this article, or claim that may be made by its manufacturer, is not guaranteed or endorsed by the publisher.

## References

[1] Benjamin EJ Wolf PA D'Agostino RB Silbershatz H Kannel WB Levy D. Impact of atrial fibrillation on the risk of death: the framingham heart study. Circulation. (1998) 98:946–52. 10.1161/01.CIR.98.10.946 9737513

[2] Lamassa M Di Carlo A Pracucci G Basile AM Trefoloni G Vanni P. Characteristics, outcome, and care of stroke associated with atrial fibrillation in Europe: data from a multicenter multinational hospital-based registry (The European Community Stroke Project). Stroke. (2001) 32:392–8. 10.1161/01.STR.32.2.392 11157172

[3] Jorgensen HS Nakayama H Reith J Raaschou HO Olsen TS. Acute stroke with atrial fibrillation. the copenhagen stroke study. Stroke. (1996) 27:1765–9. 10.1161/01.STR.27.10.1765 8841326

[4] Van den Ham HA Klungel OH Singer DE Leufkens HG van Staa TP. Comparative performance of ATRIA, CHADS2, and CHA2DS2-VASc risk scores predicting stroke in patients with atrial fibrillation: results from a national primary care database. J Am Coll Cardiol. (2015) 66:1851–9. 10.1016/j.jacc.2015.08.033 26493655

[5] Aspberg S Chang Y Atterman A Bottai M Go AS Singer DE. Comparison of the ATRIA, CHADS2, and CHA2DS2-VASc stroke risk scores in predicting ischemic stroke in a large Swedish cohort of patients with atrial fibrillation. Eur Heart J. (2016) 37:3203–10. 10.1093/eurheartj/ehw077 26941204

[6] Friberg L Rosenqvist M Lip GYH. Evaluation of risk stratification schemes for ischemic stroke and bleeding in 182 678 patients with atrial fibrillation: the Swedish Atrial Fibrillation cohort study. Eur Heart J. (2012) 33:1500–10. 10.1093/eurheartj/ehr488 22246443

[7] Shin SY Han SJ Kim JS Im SI Shim J Ahn J. Identification of markers associated with development of stroke in “clinically low-risk” atrial fibrillation patients. J Am Heart Assoc. (2019) 8:e012697. 10.1161/JAHA.119.012697 31668140 PMC6898804

[8] Kwon S Kim TJ Choi EK Ahn HJ Lee E Lee SR. Predictors of ischemic stroke for low-risk patients with atrial fibrillation: a matched case-control study. Heart Rhythm. (2021) 18:702–8. 10.1016/j.hrthm.2021.01.016 33482386

[9] Daccarett M Badger TJ Akoum N Burgon NS Mahnkopf C Vergara G. Association of left atrial fibrosis detected by delayed-enhancement magnetic resonance imaging and the risk of stroke in patients with atrial fibrillation. J Am Coll Cardiol. (2011) 57:831–8. 10.1016/j.jacc.2010.09.049 21310320 PMC3124509

[10] Akoum N Fernandez G Wilson B Mcgann C Kholmovski E Marrouche N. Association of atrial fibrosis quantified using LGE-MRI with atrial appendage thrombus and spontaneous contrast on transesophageal echocardiography in patients with atrial fibrillation. J Cardiovasc Electrophysiol. (2013) 24:1104–9. 10.1111/jce.12199 23844972 PMC3818287

[11] Rolf S Kircher S Arya A Eitel C Sommer P Richter S. Tailored atrial substrate modification based on low-voltage areas in catheter ablation of atrial fibrillation. Circ Arrhythm Electrophysiol. (2014) 7:825–33. 10.1161/CIRCEP.113.001251 25151631

[12] Müller P Maier J Dietrich JW Barth S Griese DP Schiedat F. Association between left atrial low-voltage area, serum apoptosis, and fibrosis biomarkers and incidence of silent cerebral events after catheter ablation of atrial fibrillation. J Interv Card Electrophysiol. (2015) 44:55–62. 10.1007/s10840-015-0020-6 26048130

[13] Müller P Makimoto H Dietrich JW Fochler F Nentwich K Krug J. Association of left atrial low-voltage area and thromboembolic risk in patients with atrial fibrillation. Europace. (2018) 20:359–65. 10.1093/europace/eux172 29016757

[14] Nielsen PB Skjoth F Overvad TF Larsen TB Lip GYH. Female sex is a risk modifier rather than a risk factor for stroke in atrial fibrillation: should we use a CHA_2_DS_2_-VA score rather than CHA2DS2-VASc? Circulation. (2018) 137:832–40. 10.1161/CIRCULATIONAHA.117.029081 29459469

[15] Huo Y Gaspar T Pohl M Sitzy J Richter U Neudeck S. Prevalence and predictors of low voltage zones in the left atrium in patients with atrial fibrillation. Europace. (2018) 20:956–62. 10.1093/europace/eux082 28605524

[16] Kirstein B Neudeck S Gaspar T Piorkowski J Wechselberger S Kronborg MB. Left atrial fibrosis predicts left ventricular ejection fraction response after atrial fibrillation ablation in heart failure patients: the Fibrosis-HF Study. Europace. (2020) 22:1812–21. 10.1093/europace/euaa179 32830233

[17] Fang MC Go AS Chang Y Borowsky L Pomernacki NK Singer DE. Comparison of risk stratification schemes to predict thromboembolism in people with nonvalvular atrial fibrillation. J Am Coll Cardiol. (2008) 51:810–5. 10.1016/j.jacc.2007.09.065 18294564 PMC3534960

[18] Maruyama K Uchiyama S Shiga T Iijima M Ishizuka K Hoshino T. Brain natriuretic peptide is a powerful predictor of outcome in stroke patients with atrial fibrillation. Cerebrovascu Dis Extra. (2017) 7:35–43. 10.1159/000457808 28253498 PMC5465753

[19] Ryu WS Bae EK Park SH Jeong SW Schellingerhout D Nahrendorf M. Increased left ventricular filling pressure and arterial occlusion in stroke related to atrial fibrillation. J Stroke Cerebrovasc Dis. (2018) 27:1275–82. 10.1016/j.jstrokecerebrovasdis.2017.12.009 29310958

[20] Zeng WT Sun XT Tang K Mei WY Liu LJ Xu Q. Risk of thromboembolic events in atrial fibrillation with chronic kidney disease. Stroke. (2015) 46:157–63. 10.1161/STROKEAHA.114.006881 25424480

[21] Li D Shinagawa K Pang L Leung TK Cardin S Wang Z. Effects of angiotensin-converting enzyme inhibition on the development of the atrial fibrillation substrate in dogs with ventricular tachypacing-induced congestive heart failure. Circulation. (2001) 104:2608–14. 10.1161/hc4601.099402 11714658

[22] Aimé-Sempé C Folliguet T. Rücker, Martin C Krajewska M Krajewska S. Myocardial cell death in fibrillating and dilated human right atria. J Am Coll Cardiol. (1999) 34:1577–86. 10.1016/S0735-1097(99)00382-4 10551709

[23] Fatema K Bailey KR Petty GW Meissner I Osranek M Alsaileek AA. Increased left atrial volume index: potent biomarker for first-ever ischemic stroke. Mayo Clin Proc. (2008) 83:1107–15. 10.4065/83.10.1107 18828970

[24] Pfannmüller B Boldt A Reutemann A Duerrschmidt N Krabbes-Graube S Mohr FW. Gender-specific remodeling in atrial fibrillation? Thorac Cardiovasc Surg. (2013) 61:66–73. 10.1055/s-0032-1332795 23315605

[25] Sánchez M Secades L Bordallo C Meana C Rubín JM Cantabrana B. Role of polyamines and cAMP-dependent mechanisms on 5alpha-dihydrotestosterone-elicited functional effects in isolated right atria of rat. J Cardiovasc Pharmacol. (2009) 54:310–8. 10.1097/FJC.0b013e3181b6e57f 19661811

[26] Stoddard M F Dawkins P R Prince C R Ammash NM. Left atrial appendage thrombus is not uncommon in patients with acute atrial fibrillation and a recent embolic event: a transesophageal echocardiographic study. J Am Coll Cardiol. (1995) 25:452–9. 10.1016/0735-1097(94)00396-8 7829800

[27] Hori Y Nakahara S Nishiyama N Fukuda R Ukaji T Sato H. Impact of low-voltage zones on the left atrial anterior wall on the reduction in the left atrial appendage flow velocity in persistent atrial fibrillation patients. J Interv Card Electrophysiol. (2019) 56:299–306. 10.1007/s10840-019-00532-z 30887280

[28] Shen MJ Arora R Jalife J. Atrial myopathy. JACC Basic Transl Sci. (2019) 4:640–54. 10.1016/j.jacbts.2019.05.005 31768479 PMC6872845

[29] Bisbal F Baranchuk A Braunwald E Bayés de Luna A Bayés-Genís A. Atrial failure as a clinical entity: JACC review topic of the week. J Am Coll Cardiol. (2020) 75:222-32. 10.1016/j.jacc.2019.11.013 31948652

[30] Kottkamp H. Atrial fibrillation substrate: the “unknown species”– from lone atrial fibrillation to fibrotic atrial cardiomyopathy. Heart Rhythm. (2012) 9:481–2. 10.1016/j.hrthm.2012.01.008 22245793

[31] Kottkamp H. Fibrotic atrial cardiomyopathy: a specific disease/syndrome supplying substrates for atrial fibrillation, atrial tachycardia, sinus node disease, AV node disease, and thromboembolic complications. J Cardiovasc Electrophysiol. (2012) 23:797–9. 10.1111/j.1540-8167.2012.02341.x 22554187

